# Mimotopes for Alloreactive and Conventional T Cells in a Peptide–MHC Display Library

**DOI:** 10.1371/journal.pbio.0020090

**Published:** 2004-04-13

**Authors:** Frances Crawford, Eric Huseby, Janice White, Philippa Marrack, John W Kappler

**Affiliations:** **1**Howard Hughes Medical Institute, Integrated Department of ImmunologyNational Jewish Medical and Research Center, Denver, ColoradoUnited States of America; **2**Integrated Department of Immunology, University of Colorado Health Science CenterDenver, ColoradoUnited States of America; **3**Department of Biochemistry and Molecular Genetics, University of Colorado Health Science CenterDenver, ColoradoUnited States of America; **4**Department of Pharmacology and the Program in Biomolecular Structure, University of Colorado Health Science CenterDenver, ColoradoUnited States of America

## Abstract

The use of peptide libraries for the identification and characterization of T cell antigen peptide epitopes and mimotopes has been hampered by the need to form complexes between the peptides and an appropriate MHC molecule in order to construct a complete T cell ligand. We have developed a baculovirus-based peptide library method in which the sequence encoding the peptide is embedded within the genes for the MHC molecule in the viral DNA, such that insect cells infected with virus encoding a library of different peptides each displays a unique peptide–MHC complex on its surface. We have fished in such a library with two different fluorescent soluble T cell receptors (TCRs), one highly peptide specific and the other broadly allo-MHC specific and hypothesized to be much less focused on the peptide portion of the ligand. A single peptide sequence was selected by the former αβTCR that, not unexpectedly, was highly related to the immunizing peptide. As hypothesized, the other αβTCR selected a large family of peptides, related only by a similarity to the immunizing peptide at the p5 position. These findings have implications for the relative importance of peptide and MHC in TCR ligand recognition. This display method has broad applications in T cell epitope identification and manipulation and should be useful in general in studying interactions between complex proteins.

## Abstract 2

A baculovirus expression library -- encoding peptides in the context of MHC molecules -- has been developed to identify and characterize unique peptide-MHC complexes that bind specific T cell receptors

## Introduction

The identification of peptide epitopes associated with particular αβ T cell receptors (αβTCRs) is often still a bottleneck in studying T cells and their antigenic targets in, for example, autoimmunity, hypersensitivity, and cancer. A direct genetic or biochemical attack on this problem can be successful, especially with class I major histocompatibility complex (MHCI)-presented peptides. For example, tumor (Van Der Bruggen et al. 2002) and transplantation (Scott et al. 2000; Simpson et al. 2001; Shastri et al. 2002; Sahara and Shastri 2003) peptide epitopes have been found this way. Identification of the antigenic peptide in a mix of peptides stripped from MHC molecules isolated from antigen-presenting cells (APCs) has sometimes been possible using a combination of a biological assay, peptide fractionation, and peptide sequencing (Guimezanes et al. 2001). However, this method is extremely labor intensive and depends on relatively high peptide frequency in the mix and a very sensitive bioassay. These conditions are not always achievable, especially with peptides presented by MHCII, in which peptide loading of surface MHC may require peptide concentrations orders of magnitude higher than those required for MHCI loading.

The reward for the labor involved in identifying peptide epitopes directly can often be the identification of the protein source of the peptide, especially as the sequencing of the genomes of many organisms approaches completion. However, in many situations, rather than identifying this precise peptide epitope, it is sufficient to identify a peptide “mimotope.” Mimotopes can be defined as peptides that are different in sequence from the actual peptide recognized in vivo, but that are nevertheless capable of binding to the appropriate MHC molecule to form a ligand that can be recognized by the αβTCR in question. These peptides can be very useful for studying the T cell in vitro, for altering the immunological state of the T cell in vivo (Hogquist et al. 1994), for vaccine development (Partidos 2000), and potentially in preparing multimeric fluorescent peptide–MHC complexes for tracking T cells in vivo (You et al. 2003).

Mimotopes can sometimes be identified in randomized peptide libraries that can be screened for presentation by a particular MHC molecule to the relevant T cell (Gavin et al. 1994; Linnemann et al. 2001; Sung et al. 2002; reviewed in Hiemstra et al. 2000; Liu et al. 2003). Thus far, strategies for screening these types of libraries have involved testing individual pools of peptides from the library and then either deduction of the mimotope sequence from the pattern of responses or sequential reduction in the size of the pool until a single peptide emerges. There are several limitations to this type of approach. Again, a very sensitive T cell bioassay is needed in which the activity of the correct stimulating peptide is not masked by competition with the other peptides in the pool. Also, an APC that expresses the relevant MHC molecule, but not the relevant peptide, must be found or constructed. Finally, because the screen relies on T cell stimulation, only agonist mimotope peptides are identified.

In other applications, another powerful library method has been sequential enrichment/expansion of a displayed library of protein–peptide variants by direct ligand–receptor binding, e.g., using bacterial phage or yeast (also reviewed in Liu et al. 2003). These methods have not yet been developed for the routine identification of T cell antigen mimotopes, because of the lack of a suitable system for the display of peptide–MHCs or for screening via αβTCR binding using these organisms. In this paper, we describe such a method using modifications of previously described systems for producing soluble peptide–MHC complexes (Kozono et al. 1994; Crawford et al. 1998; Rees et al. 1999) and αβTCRs (Kappler et al. 1994) from baculovirus-infected insect cells. We constructed a library of peptides displayed on the surface of baculovirus-infected cells bound to the mouse MHCII molecule, IA^b^. The peptides in the library varied in five peptide amino acids known to be surface exposed and predicted to lie within the footprint of αβTCR interaction.

Using fluorescent αβTCRs as probes, we have identified in the library mimotopes for two types of T cells, both originally produced by immunization of mice with the same IA^b^–peptide combination. One of these T cells was predicted from previous data (Liu et al. 2002) to be very dependent on all of the peptide surface exposed amino acids. Consistent with these predictions, a single peptide mimotope was identified in the library for this T cell. The sequence of this peptide was highly related to the immunizing peptide. In contrast, the other T cell was hypothesized to be very peptide promiscuous (Marrack et al. 2001) based on its broad allo-MHC reactivity. Consistent with this hypothesis, its αβTCR selected a large family of peptide mimotopes from the library. Comparison of these peptides indicated that attention of this αβTCR was focused primarily on a single position in the peptide.

## Results

### Characteristics of a Broadly Alloreactive and Conventional T Cell

**Figure 1:**
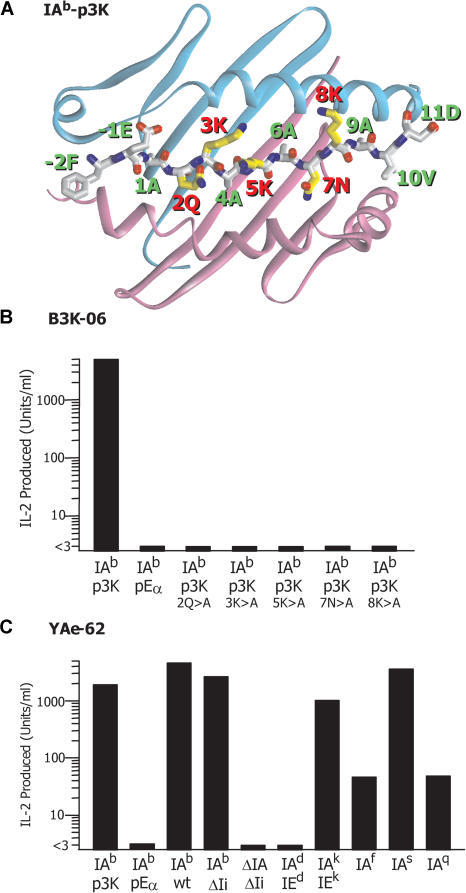
Structure of IA^b^-p3K and Properties of T Cell Hybridomas Reactive to It (A) Ribbon structure of the α1 and β1 domains of IA^b^ with a wire-frame representation of the bound p3K peptide (Liu et al. 2002). Amino acids labeled in red are the five central peptide amino acids available for αβTCR interaction. (B) The figure shows the response of 10^5^ B3K-06 hybridoma cells to various peptides presented by 10^5^ IA^b^-bearing APCs, LB-15.13. (C) The figure shows the response of the T cell hybridoma YAe-62 to various MHCII molecules. In each case, 10^5^ hybridoma cells were incubated overnight with MHCII presented in various ways. For IA^b^-p3K, soluble IA^b^-p3K was immobilized in the culture well before the addition of the hybridoma cells. In other cases, 10^6^ spleen cells were used directly as APCs without additional peptide antigen. For pEα, the spleen cells came from IA^b^-pEα/ΔIAβ/ΔIi mice (Ignatowicz et al. 1996). For wild-type IA^b^ and allo-MHCII, the spleen cells came from H-2 congenic mice on the C57BL/10 background. Finally, spleen cells from ΔIAβ/ΔIi C57BL/6 mice were used.

For this study we selected two T cell hybridomas, both prepared from IA^b^ mice immunized with the peptide p3K. This peptide binds well to IA^b^ (Rees et al. 1999), and its crystal structure bound to IA^b^ has been determined (Liu et al. 2002) (Figure 1A). The hybridoma B3K-06 was produced from wild-type C57BL/6 immunized conventionally with the peptide (Rees et al. 1999). Like most T cells resulting from immunization with a foreign peptide, it responds to IA^b^-expressing APCs in the presence, but not the absence, of p3K (Figure1B). It does not respond to APCs expressing other alleles of the IA MHCII molecule (data not shown). Also, as is commonly seen with conventional T cells, the interaction of the αβTCR of B3K-06 with IA^b^-p3K is very sensitive to changes in any of the peptide amino acids exposed on the surface of the IA^b^-p3K complex. Mutation of Q2, K3, K5, N7, or K8 to alanine virtually eliminates recognition of p3K by B3K-06 (Liu et al. 2002; see Figure 1B).

The hybridoma YAe-62 was chosen as a representative of broadly allo-reactive T cells present in mice carrying transgenes and gene knockouts that lead to expression of MHCII almost completely occupied by a single peptide (Ignatowicz et al. 1996). It was produced from IA^b^-p3K-immunized mice that express the IA^b^ molecule covalently linked to pEα, a dominant IA^b^-binding peptide derived from the MHCII IEα chain. Its properties are shown in Figure 1C. YAe-62 responds to APCs bearing IA^b^-p3K, but not to APCs lacking MHCII nor to IA^b^-pEα-bearing APCs from the mouse from which the hybridoma was derived. However, YAe-62 has additional reactivities common to many T cells isolated from these mice (Ignatowicz et al. 1996). In the absence of any added peptide, it also responds to all APCs expressing wild-type IA^b^, including those from mice with a much reduced MHCII peptide repertoire due to lack of the invariant chain. YAe-62 also responds well to APCs from a variety of mice carrying other alleles of IA. We have postulated that these T cells are focused on structural features of the MHCII molecule and are minimally dependent on direct peptide interaction (Marrack et al. 2001).

### Display of Functional Peptide–MHC on Baculovirus-Infected Insect Cells

**Figure 2:**
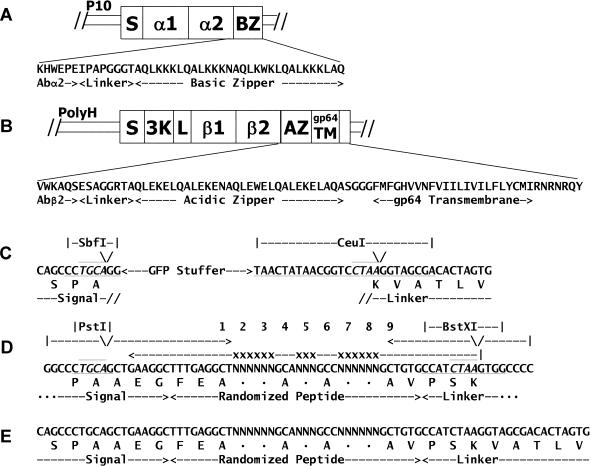
Constructions Used in These Experiments (A and B) Previously described constructions (Rees et al. 1999) for the coexpression in a single baculovirus of soluble version of the α (A) and β (B) chains of IA^b^ were modified as described in the Materials and Methods to anchor the molecule on the surface of infected insect cells. (C) The construction was further modified as described in the Materials and Methods to disrupt the IA^b^ β chain with sequence encoding enhanced GFP flanked by sites for the enzymes SbfI and CeuI. (D and E) A degenerate DNA fragment was produced by PCR (D) and cloned into the construct replacing the GFP-encoding sequence (E) as described in the Materials and Methods.

**Figure 3:**
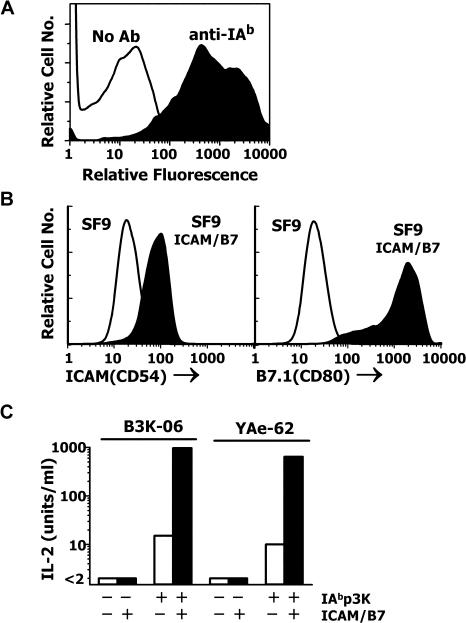
Functional Display of IA^b^-p3K on the Surface of Insect Cells (A) Sf9 insect cells were infected with baculovirus encoding a membrane-bound form of IA^b^-p3K. After 3 d, the surface expression of IA^b^-p3K was detected with an anti-IA^b^ mAb using flow cytometry. (B) The genes for mouse ICAM (CD54) and B7.1 (CD80) were cloned into an insect cell expression plasmid as described in the Materials and Methods. The plasmids were used to cotransfect Sf9 cells, and a stable transfectant (Sf9-ICAM/B7.1) was cloned expressing both proteins detected with mAbs using flow cytometry. (C) Either Sf9 (open bars) or Sf9-ICAM/B7.1 (closed bars) cells were infected with baculovirus expressing IA^b^-p3K. After 3 d, the infected insect cells were used as APCs to stimulate IL-2 production from B3K-06 and YAe-62. Uninfected cells were used as negative controls.

We previously established methods that used baculovirus-infected insect cells to produce soluble MHC molecules with covalently bound antigenic peptides (Kozono et al. 1994; Crawford et al. 1998; Rees et al. 1999). These constructions were the starting point for developing insect cells displaying functional peptide–MHCIIs. Several modifications were made to constructs that encoded the mouse MHCII molecule, IA^b^, with various bound peptides. First, to increase the stability of the molecule, an acid–base leucine zipper (O'Shea et al. 1993) was attached to the C-termini of the extracellular portions of the MHC α and β chains, replacing what would normally be the transmembrane regions of these proteins. The basic half of the zipper was attached to the α chain (Figure 2A) and the acidic half to the β chain (Figure 2B). In addition, sequence encoding the transmembrane and cytoplasmic tail of the baculovirus major coat glycoprotein, gp64, was attached to the end of the acid zipper (Figure 2B). Sf9 insect cells infected with virus encoding this construction produced the MHCII molecule at a high level anchored on the cell surface via the gp64 transmembrane (Figure 3A). Also, to make Sf9 cells better APCs (Cai et al. 1996), we established a version transfected with the genes for mouse ICAM and B7.1 (Figure 3B). When we tested the ability of Sf9 cells displaying the IA^b^-p3K complex to present the antigen to B3K-06 or YAe-62, the presence of ICAM/B7.1 greatly improved IL-2 production (Figure 3C). These results showed that IA^b^-p3K could be displayed on the surface of insect cells in a form easily recognized by T cells. In all of the experiments described below, infected conventional Sf9 cells were used for flow cytometry and infected ICAM/B7.1-expressing Sf9 cells were used in IL-2 stimulation assays.

### Detection of Displayed Peptide–MHC with Multimeric αβTCR

**Figure 4:**
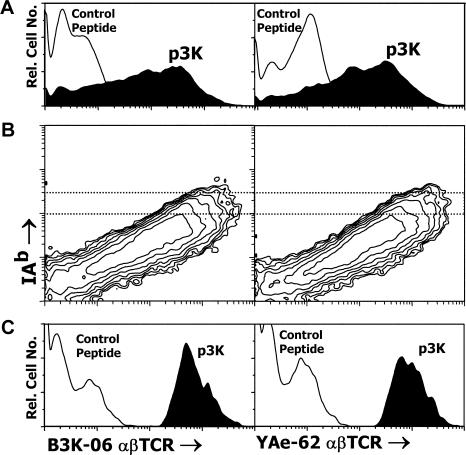
Detection of IA^b^-p3K-Expressing Insect Cells with Polyvalent, Fluorescent αβTCRs (A) Sf9 insect cells were infected with baculovirus encoding IA^b^ bound either to p3K (filled histogram) or a control peptide (FEAPVAAALHAV) (unfilled histogram). After 3 d, the infected insect cells were incubated with polyvalent, fluorescent soluble αβTCRs from B3K-06 or YAe-62. The binding of each αβTCR was assessed by flow cytometry. (B) Cells, prepared as in (A), were simultaneously analyzed with fluorescent αβTCRs and a mAb specific for IA^b^ (17–227) that does not interfere with αβTCR–IA^b^ interaction. (C) The binding of the αβTCRs is shown only for those infected insect cells that bear a high level of surface IA^b^ (dotted region in [B]).

Next we prepared fluorescent, soluble αβTCR reagents for use in flow cytometry to detect insect cells displaying the appropriate peptide–MHCII combination. Fluorescent multivalent versions of the soluble αβTCRs of B3K-06 and YAe-62 bound to insect cells displaying the IA^b^-p3K, but not a control peptide–MHCII combination (Figure 4A).

Insect cells displaying IA^b^-p3K bound the αβTCR reagents very heterogeneously (Figure 4A), probably owing to heterogeneous expression of IA^b^-p3K due to variations in the multiplicity of infection (MOI) and the lack of synchrony in viral infection and expression. To focus on cells bearing a particular level of IA^b^, we stained the cells simultaneously with the fluorescent αβTCR reagents and with an anti-IA^b^ monoclonal antibody (mAb) that did not interfere with αβTCR binding. In this case, there was a direct correlation between the amount of surface IA^b^-p3K expressed by an individual insect cell and the amount of αβTCR bound (Figure 4B) with cells bearing a particular level of IA^b^-p3K, binding the αβTCRs uniformly (Figure 4C). Therefore, comparing the two types of staining gave us a useful tool to evaluate the relation between peptide sequence and the strength of αβTCR binding (see below).

### Recovering Baculovirus Carrying a Particular Peptide–MHC Combination

**Figure 5:**
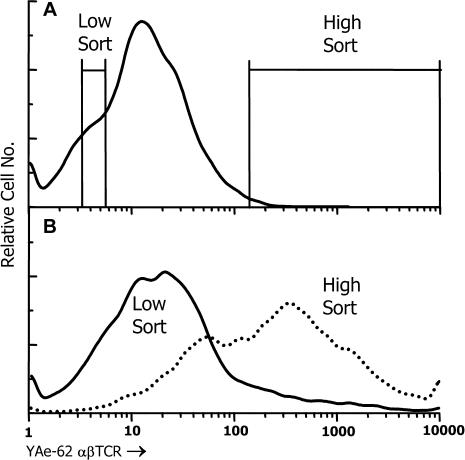
Recovery of IA^b^-p3K Virus-Infected Cells with Fluorescent αβTCR (A) Sf9 cells were infected with a mixture of virus, 99% of which encoded a control protein (a TCR β chain linked to the gp64 transmembrane/cytoplasmic tail) and 1% of which encoded IA^b^-p3K. After 3 d, the infected cells were analyzed as in Figure 3A for binding fluorescent αβTCR from YAe-62. The 1% of the infected cells with the brightest fluorescence was sorted (high sort, 15,700 cells). As a control, a similar number of cells that fluoresced as dully as the background fluorescence were also sorted (low sort). (B) The sorted cells were incubated with fresh Sf9 insect cells to allow propagation of the viruses and production of new stocks. The stocks were used to infect new Sf9 cells, and after 3 d the analysis of αβTCR binding was repeated.

Our experiments showed that fluorescent αβTCRs could be used with flow cytometry to identify insect cells infected with a baculovirus encoding a specific peptide–MHC combination. We next tested whether this system could be used to enrich baculoviruses encoding a particular peptide–MHC. Insect cells were infected at an MOI of about 1 with a mixture of baculoviruses. Of these viruses, 1% encoded the IA^b^-p3K molecule and 99% encoded a control molecule (an αβTCR β chain). The infected cells were stained with fluorescent YAe-62 αβTCR and analyzed by flow cytometry. Although a distinct population of brightly fluorescent cells was not seen, the 1% of the cells with the brightest fluorescence were sorted, as were an equal number of cells that were very dully fluorescent (Figure 5A). The recovered infected cells were cultured with fresh insect cells to produce new viral stocks. These stocks were used to infect insect cells that were tested again with the fluorescent αβTCR reagent (Figure 5B). The cells infected with virus from the few fluorescent positive cells in the original population were now nearly all brightly fluorescent, and those infected with the virus from the fluorescently dull cells were nearly all negative for binding of the αβTCR. These results showed that flow cytometry could be used with a fluorescent multimerized αβTCR to find and greatly enrich insect cells infected with a virus encoding a specific peptide–MHC combination.

### Construction of a Peptide Library Attached to IA^b^ in Baculovirus

The most widely used method for introducing gene constructions into baculovirus involves assembling the construct first in an Escherichia coli transfer plasmid, where it is flanked by sections of baculovirus DNA. The complete construct is then introduced into baculovirus by homologous recombination using any of the commercially available modified baculovirus DNAs that require homologous recombination with the plasmid in order to generate functional circular viral DNA (Kitts and Possee 1993). Based on this procedure, we constructed an IA^b^–peptide library in two steps. In the original transfer plasmid that encoded the displayed IA^b^-p3K, we flanked the site encoding the peptide with unique restriction sites, one in the section encoding the β chain leader and the other in the section encoding the linker from the peptide to the N-terminus of the β chain. The DNA between these sites was replaced with DNA encoding enhanced green fluorescent protein (GFP) (Clontech, Palo Alto, California, United States) in-frame with the IA^b^ signal peptide and with a 3′ termination codon (see Figure 2C). Thus, cells infected with baculovirus carrying this construct produced GFP, but not an IA^b^ molecule, because of disruption of the IA^b^ β chain gene.

We then designed a peptide library based on the structure of p3K bound to IA^b^ (see Figure 1A) We used oligonucleotides with random nucleotides in codons encoding five peptide amino acids (p2, p3, p5, p7, and p8) corresponding to the central surface-exposed amino acids of p3K bound to IA^b^. Other positions were kept identical to p3K, including alanines at the four standard anchor residues at p1, p4, p6, and p9. These oligonucleotides were used in a PCR to create a DNA fragment randomized in these five codons and with 5′- and 3′-end restriction enzyme sites compatible with those in the signal peptide and linker (see Figure 2D). This fragment was ligated into the restricted plasmid, replacing the GFP sequence and restoring a functional IA^b^ β chain gene (see Figure 2E). The mixture of plasmids was then used to transform E. coli and a bulk plasmid preparation was made. The plasmids were cotransfected with BaculoGold baculovirus DNA into Sf9 insect cells to produce a mixed viral stock in which each virus carried the genes for IA^b^ with a different peptide bound. Although it is difficult to calculate the efficiency with which recombination yields infectious baculovirus, we estimate the size of this library was between 3 × 10^4^ and 1 × 10^5^ independent viruses.

### Successive Enrichment of Baculovirus Carrying Peptide–MHC Combinations That Bind a Particular αβTCR

**Figure 6:**
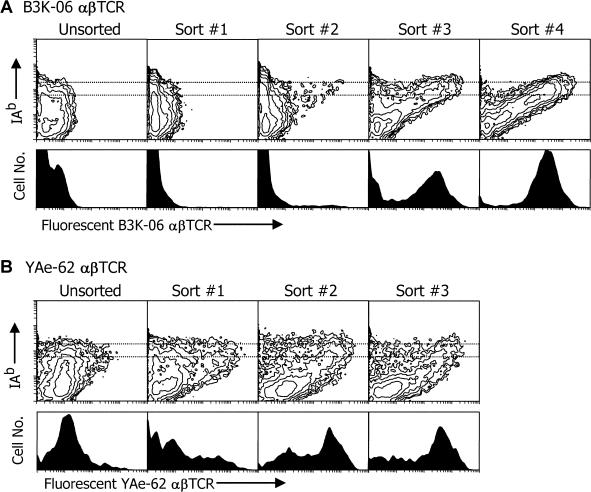
Summary of Successive Screening of the IA^b^–Peptide Libraries with Fluorescent αβTCRs Sf9 insect cells (1 × 10^7^ to 1.5 × 10^7)^ were infected at a MOI of approximately 1 with an aliquot of baculovirus encoding the IA^b^–peptide library. After 3 d, the infected cells were analyzed for binding the αβTCR of either B3K-06 or YAe-62. Either obviously fluorescent cells or the brightest 1% of the cells were sorted (2 × 10^4^ to 8 × 10^4^ cells) and added to 3 × 10^6^ fresh Sf9 cells to propagate and reexpress the viruses contained in the sorted cells. These infected cells were then reanalyzed and sorted using the fluorescent αβTCRs. This process was repeated until no further enrichment of αβTCR binding was seen. In most cases, the reanalysis was done directly from the cells that were cocultured with the sorted cells. In a few cases, an intermediate viral stock was made and then used to infect additional Sf9 cells. The turn around time per cycle was 4–7 d. The figure shows the reanalysis in a single experiment of the initial viral stocks and all of the various intermediate enriched viral stocks. Sf9 cells were infected at an MOI of less than 1 with the viral stocks and analyzed as in Figure 4 for either B3K-06 (A) or YAe-62 (B) αβTCR binding.

A large number of Sf9 insect cells were infected at an MOI of about 1, with baculovirus carrying the IA^b^–peptide library. After 3–4 d, the cells were analyzed with fluorescent B3K-06- or YAe-62-soluble αβTCR, as described above. Fluorescent cells were sorted and cultured with fresh uninfected Sf9 cells to create new infected cells for analysis and an enriched viral stock. This process was repeated three to four times. In each case, when no clear fluorescent population was apparent, the brightest 1% of the infected cells was sorted. In later rounds the majority of the cells in a clearly distinguishable fluorescent population were sorted. Figure 6 summarizes the successive enrichment of viruses that produced IA^b^–peptide combinations that could be detected with each of the fluorescent αβTCRs. Infected cells binding the B3K-06 αβTCR were apparent only after two rounds of enrichment, but eventually yielded a population with uniform binding (Figure 6A). Infected cells that bound the YAe-62 αβTCR were detectable even with the initial library of viruses and enriched rapidly to yield a population with more heterogeneous levels of binding to the receptor (Figure 6B).

### Comparison of αβTCR-Selected Peptides from the Library

**Figure 7:**
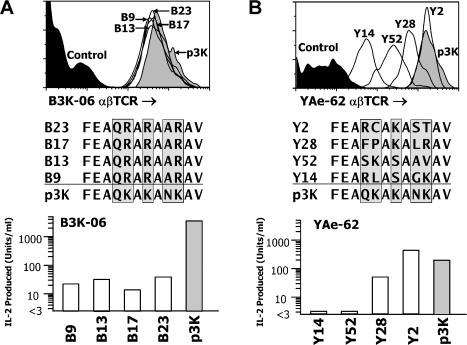
Analysis of Baculovirus Clones from the αβTCR-Enriched IA^b^–Peptide Library (A) Sf9 cells were infected with stock from four baculovirus clones (B9, B13, B17, and B23) isolated from the virus pool enriched with the αβTCR of B3K-06. After 3 d, an aliquot of cells from each infection was analyzed as in Figure 4 to assure uniform binding of the fluorescent B3K-06 αβTCR (top). Viral DNAs prepared from other aliquots of the cells were used as templates in a PCR with oligonucleotides that flanked the DNA encoding the IA^b^-bound peptide. The fragment was sequenced directly with a third internal oligonucleotide (middle). The clone stock was then used to infect Sf9-ICAM/B7.1 cells. After 3 d, the infected cells were used as APCs for B3K-06 production of IL-2 (bottom). Virus encoding IA^b^-p3K was used as a positive control. Virus encoding pEα was used as the negative control. (B) Same as (A), but using YAe-62 and clones (Y2, Y14, Y28, Y52) derived from the IA^b^–peptide library using the YAe-62 αβTCR.

At the time of the final enrichment, single infected cells binding each of αβTCRs were sorted into individual wells of 96-well culture plates containing fresh Sf9 cells in order to prepare clonal viral stocks. These stocks were used to infect fresh Sf9 cells, which were reanalyzed for binding to the appropriate αβTCR as in Figure 4. Viral DNA from the clones that showed homogeneous TCR binding at a particular level of IA^b^ were used as template in a PCR using oligonucleotides that flanked the peptide site in the construct, and a third internal oligonucleotide was used to sequence the PCR fragment. The majority of PCR fragments yielded a single unambiguous peptide sequence. These viruses were used to infect Sf9 cells that expressed mouse ICAM and B7.1. The infected cells were used as APCs for either the B3K-06 or YAe-62 hybridoma, with IL-2 production being a measure of IA^b^–peptide recognition. Viruses expressing IA^b^–peptide combinations that produced high levels of surface IA^b^, but that neither bound to the αβTCR nor stimulated the T cell hybridomas, were used as negative controls, and virus producing IA^b^-p3K was used as the positive control. Results with a few representative virus clones are shown in Figure 7A and 7B, and a summary of all of the results is shown in Table 1.

**Table 1:**
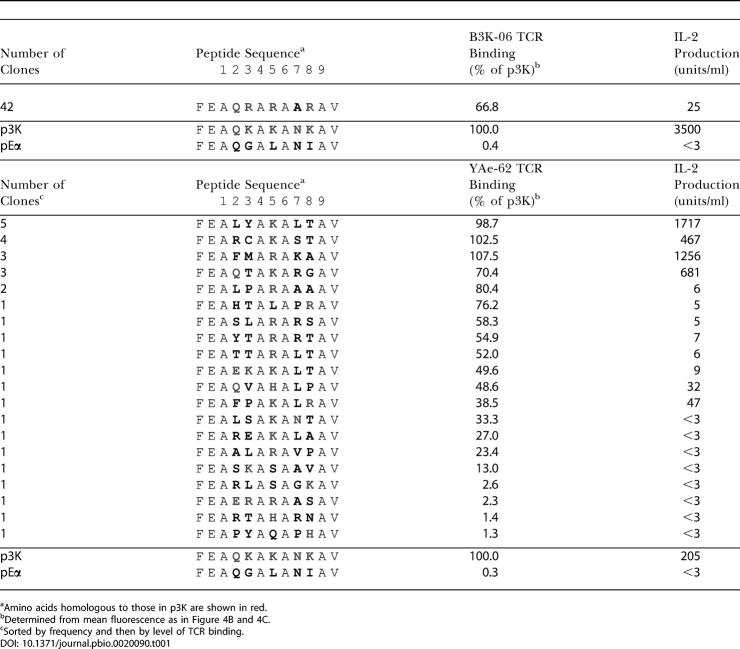
Summary of Peptides Selected by p3K-Reactive αβTCRs

^a^Amino acids homologous to those in p3K are shown in red

^b^Determined from mean fluorescence as in Figure 4B and 4C

^c^Sorted by frequency and then by level of TCR binding

Given our previous data indicating that the B3K-06 αβTCR interacted with all five of the p3K amino acids varied in this library (Liu et al. 2002; see also Figure 1B), we expected that mimotopes satisfying this receptor would be infrequent or perhaps even absent in a library of this size. Indeed, only one peptide was recovered from the library with the B3K-06 αβTCR, FEAQRARAARVD. It was found in all 42 clones analyzed with unambiguous αβTCR binding and peptide sequences. The sequence of this peptide was strikingly similar to that of p3K. Like p3K, it had a glutamine at p2. It had arginines at positions p3, p5, and p8, corresponding to the lysines found in these positions in p3K, most likely reflecting the importance of the positive charges at these positions. We do not know the relative importance of lysine versus arginine at these three positions, but given that there are two codons for lysine and six for arginine, there was of course a much higher probability of finding arginine than lysine. The most significant difference between this peptide and p3K was an alanine instead of asparagine found at p7.

When bound to IA^b^ on ICAM/ B7.1-expressing Sf9 APCs, FEAQRARAARVD was able to stimulate B3K-06 to produce IL-2, but not nearly as well as did p3K. This loss of stimulating activity was caused by one or more of the lysine-to-arginine substitutions and/or the asparagine-to-alanine substitution at p7. Interestingly, the substitution of alanine for asparagine in p3K eliminated the response of B3K-06 to soluble peptide presented by an IA^b^-bearing mouse APC (see Figure 1B). Perhaps the very high density of IA^b^–peptide on the surface of the insect cells allows for responses to peptides that would normally not be stimulatory with peptides presented by conventional APCs, although another possibility is that somehow the arginine (particularly at p8) compensated for the absence of the asparagine sidechain.

Consistent with the hypothesis that the αβTCR of YAe-62 would be more peptide promiscuous than that of B3K-06, we found 20 different peptide sequences among the analyzed clones that produced an IA^b^–peptide combination that bound the YAe-62 αβTCR. It is likely that many more would be identified if more clones were analyzed. Five sequences were found multiple times. Not unexpectedly, these were among those that bound the YAe-62 αβTCR most strongly. There was a 100-fold range in the intensity of αβTCR binding to the different IA^b^–peptide combinations, ranging from about 4-fold to 400-fold binding above that seen with a negative control peptide. One obvious property of these peptides stands out. There appeared to be a very strong selection for a basic amino acid at position 5. In 16 of 20 of the peptides, a lysine, arginine, or histidine was found at position 5, matching the lysine found in p3K. As a control, we sequenced random clones picked either from the original E. coli construction of the library (17 clones) or from the baculovirus library that expressed IA^b^–peptide well, but did not bind either αβTCR (11 clones). The frequencies of basic amino acids at p5 in these sequences were only 12% and 9%, respectively (data not shown).

There was no strong selection for amino acids homologous to those of p3K at positions p2, p3, p7, or p8. The amino acids at positions p2 and p3 appear nearly random, suggesting little or no essential contact between this part of the peptide–MHC ligand and the receptor, although these positions may contribute to the wide range of apparent αβTCR affinities seen. While not homologous to the asparagine in p3K, leucine was found at p7 in six of 20 (30.0%) of the YAe-62 αβTCR-selected peptides and three of 11 (27.2%) of the IA^b^-binding peptides that were not bound by the YAe-62 αβTCR, but only two of 17 (11.8%) of the random E. coli plasmids. The amino acid in this position is only partially exposed on the surface and can contribute significantly to peptide–MHC interaction (Liu et al. 2002). After asparagine, leucine is the most common amino acid found at this position in peptides found naturally bound to IA^b^ (Dongre et al. 2001; Liu et al. 2002). Therefore, although more data would be required to test its significance, there may have been some slight enrichment of leucine at p7 in the expressed library prior to αβTCR selection, reflecting the role of p7 in stable peptide binding to IA^b^.

The amino acid at position p8 is predicted to be fully surface exposed. In the selected peptides, rather than an amino acid homologous to the lysine of p3K, there may be an overrepresentation of amino acids with small neutral sidechains (threonine, serine, alanine, glycine) at this position. Perhaps this indicates that, in general, larger sidechains can be inhibitory at this position, but again more data would be required to test this idea.

The 12 IA^b^–peptide combinations that bound the YAe-62 αβTCR most strongly were also the ones that were able to induce IL-2 production from YAe-62. Among these, a number with the very highest apparent affinities stimulated YAe-62 better than did p3K. However, there was not a direct correlation between apparent affinity and the level of IL-2 production; i.e., several peptides that yielded complexes with IA^b^ with about the same apparent affinity for the αβTCR nevertheless stimulated very different levels of IL-2 production from YAe-62. This may be related to the phenomenon of altered peptide ligands (see Discussion).

Overall, our results supported our original prediction that for conventional T cells, such as B3K-06, most of the surface-exposed residues of the peptide would be important in peptide–MHC recognition, while for broadly allo-MHC-reactive T cells, such as YAe-62, peptide recognition would be much more promiscuous.

## Discussion

The peptide degeneracy allowed for a given αβTCR–MHC combination has been a subject of study over many years. While minor changes in the exposed amino acids sidechains of the peptide can often destroy αβTCR recognition, usually at least some variation is tolerated within the predicted footprint of the αβTCR on the peptide–MHC ligand (Evavold and Allen 1992; Reay et al. 1994). We can understand this flexibility to some extent from the X-ray structures of αβTCR–MHC–peptide complexes that show poor or even absent interactions between some peptide amino acid sidechains and the complementarity region (CDR) loops of the receptor (reviewed in Garcia et al. 1999).

We have reported the properties of mice that have been genetically manipulated to express their MHCII molecules virtually completely occupied by a single peptide (Ignatowicz et al. 1996; Marrack et al. 2001). One of the most unusual features of the repertoire of T cells that develop in these animals is that they show an unusually high frequency of broadly allo-MHC–self-MHC-reactive T cells. These T cells are lost when these animals are repopulated with MHCII wild-type bone marrow cells. We have concluded that cells of this type are commonly positively selected in normal animals, but to a large extent negatively selected by self-MHC occupied by a variety of self-peptides. Their survival in single peptide–MHC mice may reflect the need for many different peptides to expose all MHC amino acids and their various conformers during T cell-negative selection. We have proposed that the αβTCRs of these cells are focused on the common conserved features of peptide–MHC complexes rather than on the specific sidechains of the exposed amino acids of the peptide (Marrack et al. 2001). A consequence of this hypothesis is the prediction that T cells of this sort should be much more peptide promiscuous than conventional T cells.

The experiments reported here were designed to test this prediction by comparing the peptide promiscuity of one of these broadly allo-reactive T cells, YAe-62, typical of T cells from these mice, to that of a T cell with the same nominal specificity produced by immunization of conventional mice. The results support the conclusion that the broadly allo-reactive T cell has a much greater peptide promiscuity than does the conventional T cell. This question of T cell promiscuity is an important one in that it addresses the existence of a very large set of TCRs that apparently make it through positive selection, but never see the light of day in normal animals, because they are negatively selected on self-MHC with little input from the MHC-bound peptide. Thus, studying the peripheral fully negatively selected T cell repertoire gives a false impression of the interaction requirements necessary or sufficient for positive selection. These promiscuous T cells may also give us insight into possible evolutionary conserved αβTCR–MHC interactions that have been hard to sort out with conventional T cells.

While perhaps much less frequent than in single peptide–MHC mice, peptide-promiscuous T cells have been described in normal individuals (Brock et al. 1996). Consistent with the idea that this property may be linked to allo-MHC reactivity, in a parallel study we have shown that peptide-promiscuous T cells are enriched in normal mice in the population of T cells reactive to foreign MHC alleles and isotypes (Huseby et al. 2003).

In order to study the relationship between peptide sequence and αβTCR recognition, we developed a baculovirus-based display method for rapid identification of peptides that form complexes with MHC that bind a particular αβTCR. Display is one of the most powerful library techniques available. Its underlying principle is that the protein or peptide members of the library are expressed on the surface of organisms that harbor the DNA encoding them. A binding assay that isolates all members of the library with the appropriate properties copurifies the organism and the encoding DNA. The DNA is then amplified and reexpressed and the process repeated as many time as necessary to enrich fully the relevant molecules, whose sequence can be deduced from the copurified DNA. The great advantage of display libraries is that all members of the library that satisfy the screening conditions are enriched simultaneously without the need to identify them one by one.

In order for peptides to be tested for αβTCR binding, they must be complexed with the relevant MHC molecule on a platform suitable for interaction with the T cell and/or its αβTCR. For display libraries, one aspect of this problem has been solved by the ability to express MHC molecules with sequence for a covalently attached antigenic peptide imbedded in the MHC genes (Kozono et al. 1994; Mottez et al. 1995; Uger and Barber 1998; White et al. 1999). However, the most commonly used bacterial display systems do not yet allow for the assembly and display of complex multichain MHC molecules. There is a recent report of the successful display of a single-chain peptide–MHCI on yeast cells (Brophy et al. 2003), but our own previous attempts with yeast had failed to yield displayed peptide–MHCII in a form capable of recognition by T cell hybridomas (data not shown). Our previous success with producing soluble MHC and αβTCR molecules using a baculovirus expression system and a report of peptide libraries displayed in baculovirus (Ernst et al. 1998) led us to adapt these methods for surface display of peptide–MHCII on insect cells. We constructed a library of peptides attached to the displayed mouse class II molecule, IA^b^. Using fluorescently labeled multimeric soluble αβTCRs as bait and insect cells infected with the IA^b^–peptide library as fish, we were able to identify rapidly the members of the library that encoded peptide mimotopes for two αβTCRs.

In these studies, the immunizing peptide (epitope) for the αβTCR was already known. However, this method should be useful as well in identifying mimotopes for αβTCRs whose peptide epitope is not known, provided that suitable peptide anchor residues for MHC binding are known. One limitation of this display method as presented here is the size of the peptide library. The bottlenecks caused by the preparation of the library in an E. coli plasmid and then its introduction into baculovirus by homologous recombination resulted in a library with only 3 × 10^4^ to 1 × 10^5^ members. This is far below the size required to have all 3.2 × 10^6^ versions of the peptide present when randomizing five amino acids. Large libraries of this size require more efficient baculovirus-cloning methods, such as incorporation of DNA fragments directly into baculovirus DNA by ligation (Ernst et al. 1994) or in vitro recombinase-mediated recombination (Peakman et al. 1992). In preliminary experiments, we have constructed an IA^b^–peptide library with over 10^7^ clones by directly ligating (Ernst et al. 1994) a randomized PCR DNA fragment encoding the peptide into linearized baculovirus DNA using unique homing restriction enzyme (SceI–CeuI) sites introduced flanking the peptide-encoding region of the construct (data not shown). Since recircularized baculovirus DNA is directly infectious when introduced into insect cells by transfection, there is no theoretical reason why this method cannot be used to create libraries as large as those reported for yeast and phage.

We have developed this method using IA^b^ as the displayed MHCII molecule carrying the peptide library. However, using the same strategy, we have successfully displayed numerous other MHCII molecules, such as murine IE^k^ and human DR4, DR52c, and DP2 (data not shown). While the leucine zippers that we included in this construct are not strictly required for expression of IA^b^, they have helped considerably in expression of some of these other MHCII molecules. Moreover, we (White et al. 1999) and others (Mottez et al. 1995; Uger and Barber 1998) have shown that peptides can be tethered to MHCI molecules via the N-terminus of either β2m or the heavy chain, making this approach feasible for searching for MHCI-bound peptide mimotopes as well. In preliminary experiments we have successfully displayed on the surface of Sf9 cells the mouse MHCI molecule, D^d^, with a β2m-tethered peptide from HIV gp120 (data not shown). Given that baculovirus has been such a successful expression system for many different types of complex eukaryotic proteins that express or assemble poorly in E. coli, this method may have broad applications to other receptor–ligand systems.

As opposed to methods that use T cell activation as the peptide-screening method, an advantage of display methods that use flow cytometry for screening and enrichment is that the strength of binding of receptor and ligand can be estimated and manipulated. In the results reported here, by limiting the analysis to insect cells bearing a particular level of peptide–MHC, a uniform level of αβTCR binding was seen for an individual peptide sequence, but the strength of binding varied over two orders of magnitude for different peptides, presumably reflecting the relative affinity of the receptor for different IA^b^–peptide combinations. Thus, depending on whether one was interested in high- or low-affinity ligands for the αβTCR, one could enrich for peptides with these properties directly during the screening of the library. Such an approach has been used with antibody (Boder and Wittrup 2000) and αβTCR (Shusta et al. 2000) variants displayed on yeast to select directly for receptors with increased affinity.

It is worth noting that there was not a direct correlation between the strength of αβTCR binding to a particular peptide–MHC combination and the subsequent level of IL-2 secretion seen from the T cell responding to this combination. While in general the best IL-2 secretion was obtained with complexes with the highest apparent affinities, some IA^b^–peptide combinations with apparent high affinity stimulated IL-2 production poorly. One interesting possibility is that this observation is related to the phenomenon of altered peptide ligands in which amino acid variants of fully immunogenic peptides only partially activate or even anergize the T cell (Evavold et al. 1993), despite minor differences in affinity. In some cases, this phenomenon has been related to αβTCR binding kinetics, rather than just overall affinity (Lyons et al. 1996). Future experiments using surface plasmon resonance or fluorescence peptide–MHC multimers might help to test this idea.

In summary, the very properties that have made baculovirus a very successful expression system for complex eukaryotic proteins also make it suitable for library display methods, with potential application not only in T cell epitope/mimotope discovery, characterization, and manipulation, but also in studying a wide variety of other protein–protein interactions.

## Materials and Methods

### 

#### Synthetic peptides, oligonucleotides, and DNA sequencing

The peptides pEα (FEAQGALANIAVD), p3K (FEAQKAKANKAVD), and various alanine-substituted variants of p3K were synthesized in the Molecular Resource Center of the National Jewish Medical and Research Center (Denver, Colorado, United States), as were all oligonucleotides used in PCR and DNA sequencing. Automated DNA sequencing was also performed in this facility.

#### Cell lines and T cell hybridomas

The insect cell lines Sf9 and High Five were obtained from Invitrogen (Carlsbad, California, United States). The IA^b^-p3K-reactive T cell hybridoma B3K-06 was produced from C57BL/6 mice as previously described (Rees et al. 1999). The IA^b^-expressing B cell hybridoma LB-15.13 (Kappler et al. 1982) was used to present soluble peptides to B3K-06.

The T cell hybridoma YAe-62 (Marrack et al. 2001) was produced from previously described (Ignatowicz et al. 1996) C57BL/6 mice that lacked expression of the endogenous IA^b^ β gene (ΔIAβ) and the invariant chain (ΔIi) and that carried a transgene for the IA^b^ β gene that was modified to insert sequence encoding pEα and a flexible linker between the signal peptide and the N-terminus of the β chain. These mice were immunized intravenously with 3 × 10^6^ dendritic cells from ΔIAβ/ΔIi C57BL/6 mice. These cells had been retrovirally transduced (Mitchell et al. 2001; Schaefer et al. 2001) with the IA^b^ β gene that was modified as above to express with a tethered p3K. T cells from these immunized mice were propagated in vitro and converted to T cell hybridomas, by standard techniques (White et al. 2000). The hybridomas were initially screened for binding of multivalent, fluorescent IA^b^-p3K (Crawford et al. 1998; Rees et al. 1999) and subsequently for IL-2 production in response to immobilized, soluble IA^b^-p3K, but not to spleen cells from the host ΔIAβ/ΔIi IA^b^-pEα transgenic mice. Further characterization of YAe-62 is described in the Results.

#### Soluble αβTCRs

cDNA, prepared from B3K-06 and YAe-62, was used as template in a PCR using oligonucleotides that flanked the Vα and Vβ regions and introduced restriction enzyme sites that allowed cloning of the PCR fragments into a previously described baculovirus expression vector for soluble αβTCRs (Kappler et al. 1994). The cloned fragments were sequenced and incorporated into baculovirus and αβTCRs were purified from the supernatants of infected High Five cells. For B3K-06, the α chain was AV0401/AJ27 and the CDR3 sequence was CALVISNTNKVVFGTG. The β chain was BV0801/BJ0103 and the CD3 sequence was CASIDSSGNTLYFGEG. For YAe-62, the α chain was AV0412/AJ11 and the CD3 sequence was CAANSGTYQRFGTG. The β chain was BV0802/JD0204 and the CD3 sequence was CASGDFWGDTLYFGAG.

#### Expression of ICAM and B7.1 in Sf9 cells

DNA fragments encoding the baculovirus hr5 enhancer element, IE1 gene promoter, and IEI poly(A) addition region were synthesized by PCR using baculovirus DNA as template. The fragments were used to construct an insect cell expression vector (pTIE1) on a pTZ18R (Pharmacia, Uppsala, Sweden) backbone with the hr5 enhancer at the 5′-end, followed by the IE1 promoter, a large multiple cloning site (Esp3I, MunI, SalI, XhoI, BsrGI, HpaI, SpeI, BstXI, BamHI, BspEI, NotI, SacII, XbaI), and the IE1 poly(A) addition region. The complete sequence of the pTIE1 vector has been deposited in GenBank (see Supporting Information). DNA fragments encoding mouse ICAM and B7.1 were cloned between the XhoI and NotI sites of the multiple cloning site. Sf9 cells were transfected with a combination of the plasmids by the standard calcium phosphate method and cells expressing both molecules on their surfaces were cloned without selection at limiting dilution to establish the line Sf9-ICAM/B7.1.

#### IL-2 assays

T cell hybridoma cells (10^5^) were added to microtiter wells containing either (1) saturating immobilized peptide–MHC, (2) 10 μg/ml peptide plus 10^5^ LB-15.13 cells, (3) 5 × 10^4^ Sf9-ICAM/B7.1 insect cells infected 3 d previously with baculovirus encoding a displayed peptide–MHC, (4) 10^6^ spleen cells from IA^b^-pEα single peptide mice, or (5) 10^6^ spleen cells from various knockout or MHC congenic mice. After overnight incubation the culture supernatants were assayed for IL-2 as previously described (White et al. 2000).

#### mAbs and flow cytometry

The following mAbs were used in these studies: 17/227, a mouse IgG2a antibody, specific for IA^b^ (Lemke et al. 1979); ADO-304, an Armenian hamster antibody specific for an epitope on the αβTCR Cα region not accessible on the surface of T cells, but exposed on recombinant αβTCR and on CD3-dissociated, NP-40-solublized natural αβTCR (Liu et al. 1998); 3E2 (PharMingen, San Diego, California, United States), specific for mouse ICAM (CD54); and 16–10A1 (PharMingen), specific for mouse B7.1 (CD80). For flow cytometry, an unlabeled version of 17/227 was used with phycoerythrin-coupled goat anti-mouse IgG2a (Fisher Biotech, Foster City, California, United States).

To assemble multivalent fluorescent versions of the soluble αβTCRs, first a biotinylated version of ADO-304 was prepared. In brief, purified ADO-304 at 1–3 mg/ml in 0.1 M NaHCO_3_ was labeled with Sulfo-NHS-LC-Biotin (Pierce Chemical Company, Rockford, Illinois, United States) at a molar ratio of 2.5:1 (biotin:antibody) for 4 h at room temperature. The reaction was quenched with 0.1 M lysine and the product dialyzed extensively against PBS. The resulting derivative contained about one biotin per molecule of mAb. The biotinylated mAb was complexed in excess with AlexaFlour647–streptavidin (Molecular Probes, Eugene, Oregon, United States). The complex was separated from the free biotin–antibody using Superdex-200 size exclusion chromatography (Pharmacia). In preliminary experiments, the amount of soluble αβTCR required to saturate an aliquot of a large single batch of this reagent was determined. To prepare the multivalent αβTCR, the appropriate amount of soluble αβTCR was mixed with an aliquot of the fluorescent anti-Cα reagent overnight. For staining for flow cytometry, this mix was used without further purification. Each 100 μl sample contained approximately 2 μg of the fluorescent reagent plus 10^5^ Sf9 insect cells. This mixture was incubated at 27°C for 1–2 h. The cells were then washed for analysis. The advantages of this method for preparing fluorescent multimers over using direct enzymatic biotinylation (Schatz 1993) of the αβTCR were that only one fluorescent reagent needed to be prepared for all αβTCRs, the mAb–streptavidin complex was very stable over a long period of time, and no special peptide-tagged version of the soluble αβTCR was required.

Analytical flow cytometry was performed with a FacsCaliber flow cytometer (Becton-Dickinson, Palo Alto, California, United States). For sorting, a MoFlo instrument was used (Dako/Cytomation, Glostrup, Denmark).

#### IA^b^ and peptide library constructions

For displaying IA^b^ on the surface of baculovirus-infected insect cells, modifications were made as described in Figure 2A and 2B to a previously reported baculovirus construct for producing soluble IA^b^-p3K (Rees et al. 1999). Other versions of this construction were prepared encoding other IA^b^-binding peptides. The constructions were incorporated into baculovirus by homologous recombination using the BaculoGold system (PharMingen).

As described in Figure 2C, this construction was altered in the E. coli transfer plasmid to replace the portion encoding p3K with sequence encoding enhanced GFP, flanked by sites for the restriction enzymes SbfI and CeuI. A PCR fragment was produced as described in Figure 2D that encoded an IA^b^-binding peptide randomized at positions p2, p3, p5, p7, and p8, but identical to p3K at other positions. This sequence was flanked by sites for the restriction enzymes PstI and BstXI, such that the cohesive ends generated by these enzymes were compatible with those generated by SbfI and CeuI in the GFP-containing plasmid. Cloning the restricted fragment into this site regenerated a covalent peptide in-frame with the signal peptide and flexible linker of the IA^b^ β chain (see Figure 2E). After ligation of the fragment into this plasmid, a bulk transformation was performed using XL1-Blue E. coli (Stratagene, La Jolla, California, United States). An estimated 3 × 10^4^ to 10 × 10^4^ independent transformants were obtained that were used to make a mixed plasmid preparation. This mixture was incorporated into baculovirus by homologous recombination as above. In order to assure a high efficiency of conversion of plasmids to virus, 1.5 × 10^7^ Sf9 cells were cotransfected with 6 μg of the plasmid mixture and 1.5 μg of BaculoGold DNA.

## Supporting Information

### Accession Numbers

The GenBank (http://www.ncbi.nlm.nih.gov/Genbank/) accession numbers for the sequences described in this paper are B7.1 (AJ278965), baculovirus DNA (L22858), ICAM (X52264), and pTIE1 vector (AY522575).

## Abbreviations

APC: antigen-presenting cell
CDR3: third complementarity region
GFP: green fluorescent protein
ΔIAβ: inactivated class II MHC molecule IA β gene
ΔIi: inactivated class II MHC molecule invariant chain gene
mAb: monoclonal antibody
MHC: major histocompatibility complex
MHCI: class I MHC molecule
MHCII: class II MHC molecule
MOI: multiplicity of infection
p3K: a peptide containing the core sequence FEAQKAKANKAV
pEα: a peptide containing the core sequence FEAQGALANIAV
αβTCR: αβ T cell receptor
